# Myeloneuropathy in the Setting of Hypocupremia: An Overview of Copper-Related Pathophysiology

**DOI:** 10.7759/cureus.16254

**Published:** 2021-07-08

**Authors:** Dario A Marotta, Matthew C Mason, Benjamin M Abraham, Hassan Kesserwani

**Affiliations:** 1 Department of Research, Alabama College of Osteopathic Medicine, Dothan, USA; 2 Department of Neurology, Division of Neuropsychology, University of Alabama, Birmingham, USA; 3 Department of Research, Marian University College of Osteopathic Medicine, Indianapolis, USA; 4 Neurology, Flowers Medical Group, Dothan, USA

**Keywords:** copper, myelopathy, steppage gait, subacute combined degeneration, myeloneuropathy

## Abstract

A posterior cord or dorsal column myelopathy due to neurosyphilis presenting as a tabetic gait is a classic neurological vignette and is taught to all medical students. Its clinical presentation is so graphic that its simulacrum with diseases other than neurosyphilis is labeled as pseudotabes. The latter can be seen with vitamin B12 deficiency as a subacute combined degeneration (SCD) of the spinal cord, another neurology classic. However, not all cases of pseudotabes are due to posterior cord myelopathy as some can arise with other deafferentation syndromes such as polyganglioneuropathies as seen with paraneoplastic syndromes, Sjogren's syndrome, idiopathic autoimmune diseases, and post-viral neuronopathies. A unique and interesting cause of pseudotabes is due to copper deficiency; copper being a metallic trace element that is fundamental to cellular life. Herein, we present a case of copper deficiency manifesting as pseudotabes and review the biochemical properties of copper and its effects on the nervous system.

## Introduction

In biology, copper is a trace element, meaning that it is required in minute amounts for vital cellular functions. In highly metabolic tissues such as developing bone, nervous tissue, and bone marrow, small fluctuations outside of physiological ranges can have profound deleterious effects on cellular function [1]. Additionally, copper is required as a cofactor of many enzymes, including those of the mitochondrial respiratory chain (i.e., cytochrome c) and free-radical scavenging enzymes (i.e., superoxide dismutase) [2]. Copper is usually ingested in foods such as shellfish, liver, grains, nuts, legumes, and chocolate [3]. The recommended daily allowance of an adult is 0.9 milligrams (mg) per day [3]. The liver stores approximately 20 mg of copper, and the biological half-life of dietary copper ranges from 13-33 days; theoretically, a copper nutritional deficit lasting at least five half-lives (roughly 150 days) is likely to lead to symptoms of hypocupremia [4].

Copper is usually absorbed by the stomach and duodenum by the P-type adenosine triphosphatase (ATP7A) cassette, a copper-transporting enteric protein [5]. Copper is absorbed into the portal circulation, then excreted by bile, and finally into the feces. It is stored in the liver, where it is released into plasma by the copper-binding protein ceruloplasmin [2]. Urinary loss is minimal when compared to fecal loss. Zinc is also absorbed by the stomach and duodenum. Zinc induces the heavy metal-binding protein metallothionein, which avidly binds copper in the enterocyte and sheds it into the intestine for fecal excretion [5]. Therein lies the process by which zinc excess can lead to copper deficiency [5].

Interruption of any aspect of this pathway can potentially lead to copper deficiency. Malabsorption of copper can occur following gastric bypass surgery, an inflammatory enteropathy, excess use of chelators, or a nutritional deficiency [6]. A rare X-linked genetic mutation of ATP7A can lead to infantile failure to thrive, gray matter degeneration, and deformity of the occipital bone [2]. Wilson's disease (hepatocerebral degeneration) is a copper-excess syndrome caused by a mutation of the ATP7B gene required for the incorporation of copper into ceruloplasmin [2]. The failure to do so leads to the accumulation of copper in the liver and the central nervous system [7]. In an adult, a subacute combined degeneration-like phenotype and a myeloneuropathy with sensory ataxia, a "steppage gait,” has been well described [8]. Swayback is an enzootic ataxia in ruminants (i.e., cattle and sheep) that is known to lead to economic loss in the field of husbandry [9]. Histopathologically, it is associated with cavitation of cerebral and spinal cord myelin, and as a therapeutic and prophylactic measure, ruminant's diets are frequently fortified with copper [9]. The triad of sensory ataxia, leukopenia, and anemia is now a well-recognized phenotype of copper deficiency [10]. An iron-deficiency anemia may be explained by copper-deficient ceruloplasmin which oxidizes ferrous to ferric iron and reduces the capacity of transferrin to bind to iron [2]. Hephaestine, named after Hephaestus, the god of metalworking, is a copper-dependent ferroxidase allowing iron efflux from enterocytes into the systemic circulation. [10,11]. 

## Case presentation

A 65-year-old man presented to the neurology clinic with bilateral foot numbness and aching pain that ascended to his knees in a six-month period. The pain was partially controlled with gabapentin 300 mg twice daily, and he was still able to drive his truck and ambulate independently. He subsequently developed a subacute loss of balance over a period of two weeks and required assistance to stand. This was accompanied by bilateral numbness and clumsiness of his hands. No weakness of the arms or legs was reported. In particular, he reported no dysarthria, dysphagia, or diplopia. Additionally, he denied urinary or fecal incontinence and denied any orthostatic intolerance.

His past medical history was significant for hypertension, chronic low back pain, and glaucoma. His medications included amlodipine 10 mg once daily, lisinopril 40 mg once daily, gabapentin 300 mg twice daily, tramadol 50 mg as needed, and methocarbamol 500 mg twice daily. The patient had a 50-pack-year smoking history but quit in 2016 and now only utilizes electronic cigarettes. He denied unintentional weight loss, fatigue, headache, vision loss, shortness of breath, and gastrointestinal symptoms (i.e., malabsorption syndrome, intake of zinc supplements, or denture creams.)

On physical examination, the patient appeared thin but well-nourished and in no acute distress. The patient was seated in a wheelchair. He was oriented to person, place, problem, and time with appropriate mood and affect. He was unable to stand without assistance, and he had a positive Romberg sign with a steppage gait. He required assistance to walk. The patient’s gait and cadence can be seen in Video 1.

**Video 1:** Physical Examination Finding: Steppage Gait

Cranial nerve examination revealed full ocular motion with symmetric pupils, normal consensual, and accommodative reflexes. Facial muscle strength, including eye closure, smile, and buccinator function, was symmetric and normal. The gag reflex was present with no hypophonia or dysarthria. Sternocleidomastoid function with head rotation and neck flexion was normal. Shoulder shrug was also normal. Upper and lower motor strength was fully intact throughout (5/5 on the medical research council grading system). Pseudoathetosis was present with dysmetria bilaterally on finger-to-nose and heel-to-shin. Decreased vibration and joint position sense were detected in the fingers and toes. There was also a length-dependent loss to pin-prick in the feet. Patellar and Achilles deep tendon reflexes were absent bilaterally (0/4) with hyporeflexia in the upper extremities.

A nerve conduction/electromyogram of the lower extremities revealed evidence of a moderately severe axonal sensory-motor polyneuropathy. Comparatively, the nerve conduction study of the upper extremities revealed evidence of bilateral absent radial, ulnar, and sensory amplitudes with a demonstration of bilateral carpal tunnel syndrome and bilateral cubital tunnel syndrome (without conduction block). An electromyographic study of the arms and legs revealed chronic distal denervation of the legs in a typical length-dependent neuropathy manner. Of note, there was no evidence of active denervation or re-innervation (nascent motor units). The global sensory loss indicates an element of a polyganglioneuropathy in addition to the length-dependent polyneuropathy.

**Figure 1:**
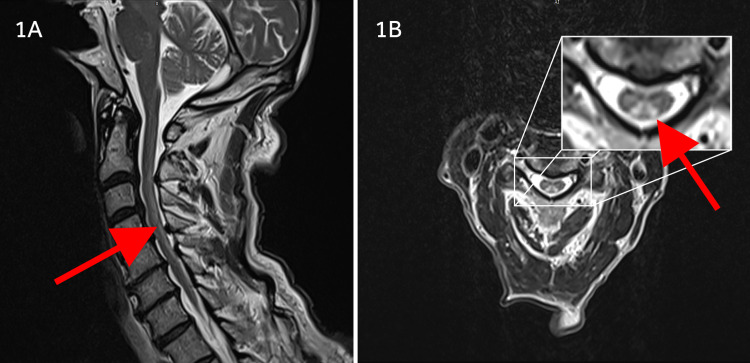
T2-weighted MRI images of the cervical cord 1A: Sagittal images revealing longitudinally extensive posterior cord demyelinative lesion (red arrow). 1B: axial cut demonstrating posterior cord lesion with inverted V orientation (red arrow). MRI=magnetic resonance imaging

Magnetic resonance imaging (MRI) of the brain without contrast revealed extensive periventricular and scattered subcortical white matter with a high T2/Fluid-attenuated inversion recovery (FLAIR) signal. Encephalomalacia was evident in the right posterior internal capsule, thalamus, and extending to the parietal corona radiata and centrum semiovale. Without contrast, MRI of the cervical spine revealed diffuse myelomalacia affecting the posterior cord extending from C2 through C7 in an inverted V fashion (shown in Figure 1). There was severe spondylosis with significant spinal stenosis and cord compression worse at C4-5. MRI imaging of the thoracic and lumbar spine was unremarkable.

A lumbar puncture was performed. Cerebral spinal fluid (CSF) showed mildly elevated protein with normal immunoglobulin G (IgG) index and no oligoclonal bands. A detailed report of CSF laboratory findings is summarized in Table 1.

**Table 1:** Summary of cerebral spinal fluid analysis results IGG=IgG index measures; quant=quantitative; WBC=white blood cell; RBC=red blood cell

Metabolite	Result	Reference Range	Units
Protein Total	63.7**	15-45	mg/dL
IGG, quant	1.9	0-8.6	mg/dL
Albumin	23	11-48	mg/dL
Immunoglobulin G, serum	771	603-1613	mg/dL
Albumin, serum	4.1	3.8-4.8	g/dL
Oligoclonal bands	0	0	
Glucose	56	40-75	mg/dL
WBC	0	No reference value	
RBC	384	No reference value	

Extensive serology studies, including a comprehensive metabolic panel (CMP), complete blood count (CBC), serum antibodies, and metal/mineral/vitamin diagnostics, were performed and are displayed in Table 2, Table 3, Table 4, and Table 5, respectively. In particular, aquaporin-4 antibodies (AQP-4) and myelin oligodendrocyte glycoprotein (MOG) antibodies were absent. In summary, the patient’s serology was significant for elevated blood urea nitrogen (BUN) and creatinine and normocytic anemia with leukocytosis. Of note, serum copper levels were low (16 ug/dL; reference range: 69-132), Vitamin E Gamma tocopherol levels were low (0.2 mg/L; reference range: 0.5-4.9), and methylmalonic acid levels were elevated (561 nmol/L; reference range: 0-378). Serum zinc levels were within normal limits. Ceruloplasmin studies were ordered, but laboratory oversight precluded pre-supplementation quantification. Ceruloplasmin measurement after copper supplementation was 20.2 mg/dL (reference range: 16-31 mg/dL).

**Table 2:** Summary of comprehensive metabolic panel results BUN=blood urea nitrogen; EGFR=estimated glomerular filtration rate; AST (SGOT)=aspartate transaminase (serum glutamic-oxaloacetic transaminase); ALT (SGPT)=alanine transaminase (serum glutamic-pyruvic transaminase)

Metabolite	Result	Reference Range	Units
Glucose	78	65-99	mg/dL
BUN	37**	8-27	mg/dL
Creatinine	1.41**	0.76-1.27	mg/dL
EGFR	52**	>59	mL/min/1.73 m^2^
BUN/Creatinine Ratio	26**	10-24	None (ratio)
Sodium	140	134-144	mmol/L
Potassium	4.5	3.5-5.2	mmol/L
Chloride	106	96-106	mmol/L
Carbon dioxide, total	23	20-29	mmol/L
Calcium	9.2	8.6-10.2	mg/dL
Alkaline phosphatase	87	39-117	IU/L
AST (SGOT)	20	0-40	IU/L
ALT (SGPT)	23	0-44	IU/L

**Table 3:** Summary of complete blood count results WBC=white blood cell; RBC=red blood cell; MCV=mean corpuscular volume; MCHC=mean corpuscular hemoglobin concentration

Metabolite	Result	Reference Range	Units
WBC	11.0**	3.4-10.8	x10E3/uL
RBC	3.61**	4.14-5.80	x10E6/uL
Hemoglobin	10.4**	13.0-17.7	g/dL
Hematocrit	33.0**	37.5-51.0	%
MCV	91	79-97	fL
MCHC	31.5	31.5-35.7	g/dL
Platelets	378	150-450	x10E3/uL
Neutrophils	77	Not established	%
Lymphocytes	16	Not established	%
Monocytes	7	Not established	%
Eosinophils	0	Not established	%
Basophils	0	Not established	%

**Table 4:** Summary of antibody serology MOG=myelin oligodrendrocyte glycoprotein; NMO=neuromyelitis optica; IFA=Immunofluorescence assay; IGG=immunoglobulin G; NA=not applicable

Metabolite	Result	Reference Range	Units
MOG antibody, cell-based IFA	Negative	Negative	NA
NMO IGG autoantibodies	<1.5	0.0-3.0	U/mL

**Table 5:** Summary of metal, mineral, and vitamin diagnostics CRT=creatinine; hr=hour

Metabolite	Result	Reference Range	Units
Copper, serum	16**	69-132	ug/dL
Copper, urine	8	Not established	ug/L
Ceruloplasmin	20.2	16-31	mg/dL
Creatinine (CRT)	0.61	0.30-3.00	g/L
Copper/creatinine ratio	13	0-49	ug/g creat
Copper, urine 24 hr	14	3-35	ug/24 hr
Homocysteine	12.7	5.0-15.0	umol/L
Methylmalonic acid, serum	561**	0-378	nmol/L
Vitamin B12	836	232-1245	pg/mL
Vitamin E (Alpha Tocopherol)	18.1	9.0-29.	mg/L
Vitamin E (Gamma tocopherol)	0.2**	0.5-4.9	mg/L
Zinc, plasma or serum	92	44-115	ug/dL

Prior to the availability of the serum copper, the patient was treated with intravenous methylprednisolone one gram daily for three days with no improvement in his symptoms. The patient was prescribed copper gluconate (2 mg *per os *(po) *bis in die* (bid) for 2 weeks, then 2 mg po once daily), Vitamin B12 (1,000 mcg sublingual daily), and Vitamin E (400 units daily). After nearly two weeks of therapy, the patient’s symptoms plateaued, and the decline was halted. Rehabilitation with physical therapy was started. He remains stable and under routine evaluation and monitoring for new and/or worsening symptoms.

## Discussion

Copper is a cofactor of many fundamental enzymes that are critical for nervous tissue health and maintenance. There are at least 14 enzymes requiring copper as a cofactor. The 10 enzymes potentially affecting nervous tissue metabolism directly are listed below. Equally important is the delivery of copper across cell membranes via chaperone proteins (Table 6) [12].

**Table 6:** Enzymes and chaperone proteins that utilize copper as a co-factor and their functions in cellular biochemistry ATP=adenosine triphosphate

Enzyme or Copper Carrier	Function
Cytochrome-c oxidase	Electron transport, cell respiration, ATP-formation; oxdative phosphorylation
Copper/zinc superoxide dismutase	Free radical scavenging: anti-oxidant
Dopamine-beta monooxygenase	Formation of catecholamine (chromaffin granules)
Prion protein	Copper sequestration and anti-oxidant properties
Tyrosinase	Formation of melanin (melanocytes)
Lysyl oxidase	Cross-linkage of collagen and elastin
Ceruloplasmin	Oxidation of Iron (II) to Iron (III): facilitation transferrin carriage of iron on transferrin
Hephaestin	Oxidation of Iron (II) to Iron (III): facilitation on apical intestinal cells allowing iron uptake by transferrin
Methionine synthetase	Transfer of methyl groups for purine synthesis

Table 6 shows that these proteins are also involved in connective tissue formation and hematopoiesis in addition to cellular respiration and neurotransmitter synthesis. A brief overview of copper-related diseases is briefly summarized below (Table 7) [12].

**Table 7:** The hallmark copper-related genetic diseases

Disease	Mutation	Physiology	Pathology
Menke's	X-linked, ATP7A - leads to copper deficiency	Reduced copper absorption and transport across the gut	Gray matter degeneration, arterial wall fragility and osteoporosis
Mild Menke's / Occipital horn syndrome	X-linked, ATP7A - leads to copper deficiency -milder variant	Reduced copper absorption and transport across the gut	Occipital horns, skeletal deformities, Ehlers-Danlos phenotype, autonomic neuropathy
Wilson's	Autosomal recessive, ATP7B	Reduced biliary excretion of copper into bile	Hepatic disease, hemolytic anemia, corneal disease, basal ganglion disease: dystonia, subcortical dementia

The clinical phenotypes can be understood by analyzing the biochemistry of elemental copper. Copper is a coenzyme of the mitochondrial enzyme cytochrome c, which is required for electron transport in the respiratory chain; thus, explaining why the metabolically active long tracts of the spinal cord (dorsal column) are particularly susceptible to the effects of copper deficiency [2]. The role of copper as a coenzyme of the free radical scavenger, superoxide dismutase, may also be partly explanatory. The kinky hair of Menke's disease and the Ehlers Danlos phenotype of the "occipital horn syndrome" can be explained by copper being a coenzyme of lysyl oxidase, which cross-links collagen and elastin in connective tissues, including bone and hair [2]. Depigmentation of hair and the skin may be explained by copper acting as a coenzyme of tyrosinase, a rate-limiting enzyme in the production of melanin [5]. The involvement of the enzyme dopamine beta-hydroxylase, which catalyzes dopamine to norepinephrine, may explain the autonomic neuropathy seen in diseases of copper deficiency [12].

Hematologically, a useful clue to copper deficiency is anemia which can be normocytic, macrocytic, or more rarely, microcytic that is unresponsive to vitamin B12 and iron supplementation [10]. This can mimic myelodysplastic anemia especially if associated with neutropenia. A bone marrow aspirate is also usually hypocellular, mimicking a myelodysplastic syndrome with a left-shift in myeloid and erythroid precursors and ringed sideroblasts [13]. Hepatic involvement in Wilson's disease can be extremely variable (i.e., asymptomatic, chronic hepatitis, end-stage liver failure, or even hepatic cancer) and may antedate the neurologic manifestations [14]. A pre-morbid history of major intestinal surgery or malabsorption syndrome may be a clue to copper deficiency, as is the use of zinc in dentures or supplements, with the former reducing and the latter antagonizing copper absorption [15,16].

A profile of SCD resistant to parenteral vitamin B12 supplementation should also raise concern. Paresthesias of the lower extremities, sensory ataxia with a steppage gait, and pseudoathetosis of the hands are frequent symptoms. Upper motor neuron signs, including long tract signs (i.e., spasticity, hyperreflexia, and neurogenic bladder), are also common manifestations. While the Lhermitte phenomenon is uncommon, it may also be suggestive in the setting of hypocupremia [17]. The polyneuropathy of copper deficiency is usually an axonal sensorimotor polyneuropathy. Despite prominent dorsal column involvement, pure motor or sensory neuropathy is rarely seen. The severity is also quite variable. Bilateral finger drop has been reported as lead neuropathy and should also be a cause for concern [18].

## Conclusions

Despite our patient's pseudotabes presentation and the narrow differential diagnosis, this case proved to be quite challenging. Given the patient’s typical clinical presentation, neuroimaging, response to copper supplementation, and exclusion of other potential etiologies, we remain cautiously optimistic with the intent of reappraising his clinical status on a frequent basis. Recovery from copper deficiency can be quite prolonged and incomplete. Nevertheless, our case report highlights the need to be vigilant about unusual causes of pseudotabes and stresses the understanding of the role of copper in nervous system activity. We also outline the copper pathophysiology as it relates to the nervous system. 

## References

[1] Bertinato J, L'Abbé MR (2004). Maintaining copper homeostasis: regulation of copper-trafficking proteins in response to copper deficiency or overload. J Nutr Biochem.

[2] Mercer JFB (2001). The molecular basis of copper transport diseases. Trends Mol Med.

[3] (2021). NIH Office of Dietary Supplements: copper fact sheet for health professionals. https://ods.od.nih.gov/factsheets/Copper-HealthProfessional/#en1.

[4] Linder MC, Hazegh-Azam M (1996). Copper biochemistry and molecular biology. Am J Clin Nutr.

[5] Peña MM, Lee J, Thiele DJ (1999). A delicate balance: homeostatic control of copper uptake and distribution. J Nutr.

[6] Sternlieb I, Janowitz HD (1964). Absorption of copper in malabsorption syndromes. J Clin Invest.

[7] Danks DM (1995). The metabolic and molecular basis of inherited diseases. https://ommbid.mhmedical.com/book.aspx?bookID=2709#225069413.

[8] Kumar N, Crum B, Petersen RC, Vernino SA, Ahlskog JE (2004). Copper deficiency myelopathy. Arch Neurol.

[9] Rizwan M, Khan OA, Sarwar M, Kashif M (2016). Swayback disease in ruminants: a review. Applied Science and Business Economics.

[10] Wazir SM, Ghobrial I (2017). Copper deficiency, a new triad: anemia, leucopenia, and myeloneuropathy. J Community Hosp Intern Med Perspect.

[11] Lazarchick J (2012). Update on anemia and neutropenia in copper deficiency. Curr Opin Hematol.

[12] Massaro EJ (2002). Handbook of Copper Pharmacology and Toxicology.

[13] Halfdanarson TR, Kumar N, Li CY, Phyliky RL, Hogan WJ (2008). Hematological manifestations of copper deficiency: a retrospective review. Eur J Haematol.

[14] Boga S, Ala A, Schilsky ML (2017). Hepatic features of Wilson disease. Handb Clin Neurol.

[15] Kumar N, McEvoy KM, Ahlskog JE (2003). Myelopathy due to copper deficiency following gastrointestinal surgery. Arch Neurol.

[16] Prodan CI, Holland NR, Wisdom PJ, Burstein SA, Bottomley SS (2002). CNS demyelination associated with copper deficiency and hyperzincemia. Neurology.

[17] Kumar N (2006). Copper deficiency myelopathy (human swayback). Mayo Clin Proc.

[18] Jaiser SR, Winston GP (2010). Copper deficiency myelopathy. J Neurol.

